# Comparison of Drug Delivery Systems with Different Types of Nanoparticles in Terms of Cellular Uptake and Responses in Human Endothelial Cells, Pericytes, and Astrocytes

**DOI:** 10.3390/ph17121567

**Published:** 2024-11-22

**Authors:** Hakan Sahin, Oguz Yucel, Paul Holloway, Eren Yildirim, Serkan Emik, Gulten Gurdag, Gamze Tanriverdi, Gozde Erkanli Senturk

**Affiliations:** 1 Department of Histology and Embryology, Cerrahpasa Faculty of Medicine, Istanbul University-Cerrahpasa, Istanbul 34098, Turkey; tgamze@iuc.edu.tr (G.T.); gozde.erkanlisenturk@iuc.edu.tr (G.E.S.); 2 Department of Chemical Engineering, Faculty of Engineering, Istanbul University-Cerrahpasa, Istanbul 34320, Turkey; oguz.yucel@iuc.edu.tr (O.Y.); eren.yildirim@iuc.edu.tr (E.Y.); serkan.emik@iuc.edu.tr (S.E.); ggurdag@iuc.edu.tr (G.G.); 3 Radcliffe Department of Medicine, University of Oxford, Oxford OX3 9DU, UK; paul.holloway@rdm.ox.ac.uk

**Keywords:** drug delivery system, poly(lactide-*co*-glycolide), bovine serum albumin, human serum albumin, nanolipid carriers, endothelial cell, pericyte, astrocyte, ultrastructure

## Abstract

**Background/Objectives**: The key components of the blood–brain barrier (BBB) are endothelial cells, pericytes, astrocytes, and the capillary basement membrane. The BBB serves as the main barrier for drug delivery to the brain and is the most restrictive endothelial barrier in the body. Nearly all large therapeutic molecules and over 90% of small-molecule drugs cannot cross the BBB. To overcome this challenge, nanotechnology, particularly drug delivery systems such as nanoparticles (NPs), have gained significant attention. **Methods**: Poly(lactide-*co*-glycolide) (PLGA) and albumin-based NPs (bovine/human), with or without transferrin (Tf) ligands (BSA, HSA, BSA-Tf, HSA-Tf), and nanolipid carriers (NLC) were synthesized. The interactions of these NPs with human brain microvascular endothelial cells (hBMECs), human brain vascular pericytes (hBVPs), and human astrocytes (hASTROs) were analyzed. **Results**: At doses of 15.62 µg/mL, 31.25 µg/mL, and 62.5 µg/mL, none of the NPs caused toxic effects on hBMECs, hBVPs, or hASTROs after 3 h of incubation. All NPs were internalized by the cells, but BSA-Tf and HSA-Tf showed significantly higher uptake in hBMECs in a dose-dependent manner. Ultrastructural analysis revealed notable differences between NP formulation and cell type. **Conclusions**: Our findings underscore the potential of ligand-targeted NPs to selectively interact with BBB endothelial cells. Ultrastructural analysis reveals distinct cellular processing pathways for various NP formulations across BBB-associated cell types, with autophagy emerging as a crucial mechanism for NP handling in pericytes and astrocytes. Changes in NP chemical properties upon biological exposure present significant challenges for nanomedicine design, emphasizing the need for further investigation into NP interactions at the cellular and subcellular levels.

## 1. Introduction

The blood–brain barrier (BBB) consists of the capillary basement membrane and three key cell types: endothelial cells, pericytes, and astrocytes. The primary functions of the BBB are to preserve brain homeostasis, protect the central nervous system (CNS) from harmful substances, and ensure the delivery of nutrients to the brain. The BBB provides a highly selective barrier by preventing the paracellular diffusion of hydrophilic molecules, facilitating the active transport of nutrients into the brain, promoting the efflux of drugs and hydrophobic molecules from the brain into the bloodstream, and regulating the transendothelial migration of pathogens and circulating immune cells. Tight junctions located between cerebral endothelial cells provide a uniquely tight cell to cell adhesion preventing paracellular aqueous diffusion between adjacent endothelial cells, thereby restricting the passive movement of proteins and polar solutes in and out of the CNS. Endothelial cells within the BBB are distinct from those found in the rest of the body, as they lack fenestrations, possess more extensive tight junctions, and exhibit limited vesicular transport. Astrocyte end-feet, which encase the vessel wall, are vital for the formation and maintenance of the tight junction barrier and enable an interface with neuronal demands through neurovascular coupling. Pericytes embedded within the capillary basement membrane also play a key role in the formation and maintenance of the BBB. These cells play a critical role in angiogenesis, maintaining the structural integrity and differentiation of microvessels, and promoting the formation of endothelial tight junctions [1,2,3].

The BBB acts as the main barrier for drug delivery to the brain, being the most restrictive endothelial barrier in the body. It is estimated that nearly all large therapeutic molecules and over 90% of small-molecule drugs are unable to cross the BBB. In adsorptive-mediated transcytosis at the BBB, the process begins with the attachment of positively charged biomolecules to the negatively charged endothelial surface, followed by internalization [4]. Thus, generally, only positively charged, lipophilic compounds with molecular weights below 400–600 Da can permeate the BBB. As a result, various specialized transport mechanisms have been developed to enable the passage of molecules across this barrier [1]. To facilitate the successful delivery of therapeutic agents across the BBB and into the CNS, researchers are currently exploring innovative approaches and methodologies. One rapidly growing field is the application of nanotechnology in medicine, particularly drug delivery systems including nanoparticles (NPs), which have gained significant attention in this context [5,6,7].

NPs are typically classified into three primary categories depending on their composition: organic, which include particles made from proteins, carbohydrates, lipids, or similar biomolecules; carbon-based, composed entirely of carbon atoms; and inorganic, which consist of materials like metals, ceramics, or semiconductors [8]. Among these, poly(lactide-*co*-glycolide) (PLGA) NPs have emerged as the predominant polymeric drug carriers and are extensively investigated for biomedical applications in clinical studies [9]. PLGA NPs have been shown to enhance the penetration of drugs across the BBB in both in vitro and in vivo studies [10]. Such brain-targeted PLGA NPs have been shown to reach CNS neurons following systemic administration, thus highlighting their significant potential as a brain drug delivery system [11,12]. Albumin-based NPs have also shown significant potential in delivering anticancer drugs to tumor sites, enhancing drug accumulation, and overcoming drug resistance. There are currently several albumin-based nanomedicines tested in clinical trials for potential use as cancer therapeutics [13]. Albumin-based NPs are frequently utilized in brain research due to their enhanced permeability across the BBB [14]. Notably, in our recent study, we found that the chronic administration of oxytocin-loaded albumin-based NPs positively affected hippocampal damage by enhancing neurogenesis, reducing apoptosis, and decreasing seizure severity [15]. Another frontrunner in drug delivery formulations is lipid NPs, and the clinical development of lipid-based NP technologies has demonstrated the potential of these carriers in the treatment of a range of diseases [16]. While lipid NPs have been extensively studied for drug delivery to peripheral organs such as muscle and the liver, their potential for delivering drugs to challenging targets like the brain requires further investigation. However, the high drug-loading capacity of these NPs makes them promising carriers for brain-targeted drug delivery. Therefore, lipid-based NPs have been developed for drug delivery and imaging in conditions such as Parkinson’s disease, Alzheimer’s disease, stroke, and glioblastoma multiforme [17].

Engineered NPs hold considerable potential to enhance treatment specificity and improve disease detection through cell-specific targeting, directed molecular transport to specific organelles, and other advanced techniques. Nanotechnology can help to overcome key challenges associated with traditional drug delivery methods, such as biodistribution, timed release, and precision cellular targeting. Notably, NPs can facilitate membrane transport, extend circulation duration, and enhance the stability and solubility of encapsulated therapeutic agents. As a fundamental approach in nanomedicine, effective therapeutic delivery aims to maximize efficacy while minimizing off-target toxicity by overcoming various pathophysiological barriers [18,19].

Transcytosis, an active transport mechanism that facilitates the direct movement of receptors and ligands across a cell, has emerged as a promising strategy to address the limitations of passive drug delivery. This ATP-dependent process, driven by electrostatic interactions or receptor-mediated endocytosis, enables the active transport of macromolecules across cellular barriers. Therefore, the identification of an enhanced transcytosis mechanism at the BBB has inspired significant research to exploit this pathway for drug delivery in brain-related diseases. A notable approach in this area is transferrin (Tf) receptor-mediated transcytosis, which facilitates the efficient delivery of therapeutic agents to the CNS. Additionally, recent research has explored various stimuli-responsive elements to activate transcytosis, creating powerful opportunities for targeted drug delivery and enhanced tissue penetration. A previous study involving different nanomaterials such as silica, titania, and albumin or Tf-conjugated gold NPs of various sizes has shown that these NPs are internalized to varying degrees in a human in vitro BBB model, with accumulation observed along the endo-lysosomal pathway. Rare instances of NP transcytosis were also noted among several tested materials [20,21,22,23].

These factors have contributed to the widespread focus on NP research, yielding promising results in vitro and in small animal models. However, despite significant research investment, the number of nanomedicines available to patients remains lower than expected. This shortfall is partly due to a translational gap between animal studies and human clinical trials [8,24]. In addition, nanomedicine is still in its early stages of development and faces several safety challenges, including unstandardized protocols and concerns about the prolonged retention of NPs in organs like the kidneys and lungs [25]. To maximize the clinical benefits of nanomedicines while minimizing the side effects, researchers need a deep understanding of NPs’ interactions at both the cellular and subcellular levels [26]. Therefore, understanding the interplay between different NP formulations and the key cell types of the human BBB is crucial for CNS-aimed treatments.

This study aims to examine the internalization and interactions of NPs on human endothelial cells, pericytes, and astrocytes. To achieve this, various formulations of NPs, including PLGA, bovine serum albumin (BSA), human serum albumin (HSA), and nanolipid carriers (NLCs), were synthesized. Subsequently, the interactions of these NPs with cells isolated from the human BBB, namely primary human microvascular endothelial cells (hBMECs), primary human brain vascular pericytes (hBVPs), and primary human astrocytes (hASTROs), were analyzed using histological investigations.

## 2. Results

### 2.1. Synthesis and Characterization of Nanoparticles for In Vitro Imaging Studies

#### 2.1.1. Particle Size and Polydispersity of Nanoparticles

The average particle sizes and polydispersity index (PDI) values of the NPs synthesized for use in in vitro imaging studies were determined by dynamic light scattering (DLS, Malvern Instrument, ZetasizerNano-ZS90, Malvern, UK) analysis. The average particle sizes of the AuNP and Zr-MOF markers were found to be 28.2 nm and 57.7 nm, respectively. The PDI values of these particles were determined as 0.135 and 0.148, respectively. These results indicate that both NP markers are appropriately sized with narrow size distributions, making them suitable for loading into NPs used in in vitro studies, such as PLGA, BSA, HSA, and NLC.

The DLS results of the synthesized NPs and their markers showed that the particle sizes of AuNP-labeled BSA and HSA NPs were 223.3 nm and 114.5 nm, respectively. The PDI values of these NPs were determined to be 0.189 and 0.228, respectively.

Similarly, the particle sizes of AuNP-labeled and Tf-conjugated BSA (BSA-Tf) and HSA (HSA-Tf) NPs were found to be 364 nm and 181.3 nm, respectively. The PDI values of these NPs were found to be 0.324 and 0.352, respectively. After Tf modification, a significant increase in particle size was observed for both NPs. Furthermore, in addition to the increase in particle size, the PDI values also increased following Tf modification, indicating that the modification processes caused interactions between NPs and increased the heterogeneity in the distribution.

**Figure 1:**
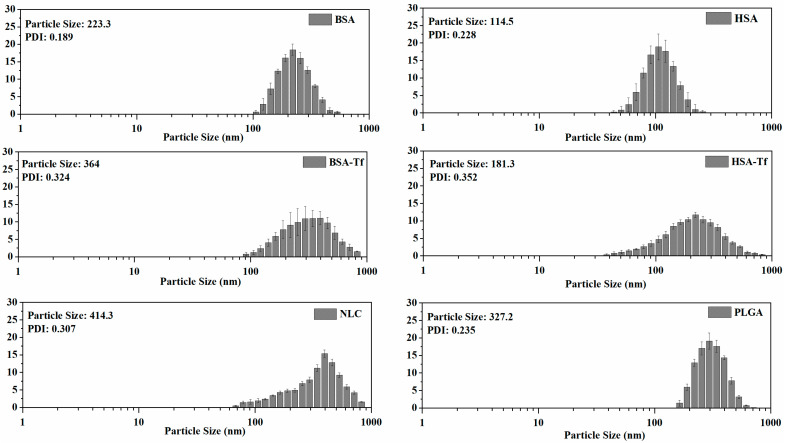
DLS measurement results of synthesized NPs.

**Figure 2:**
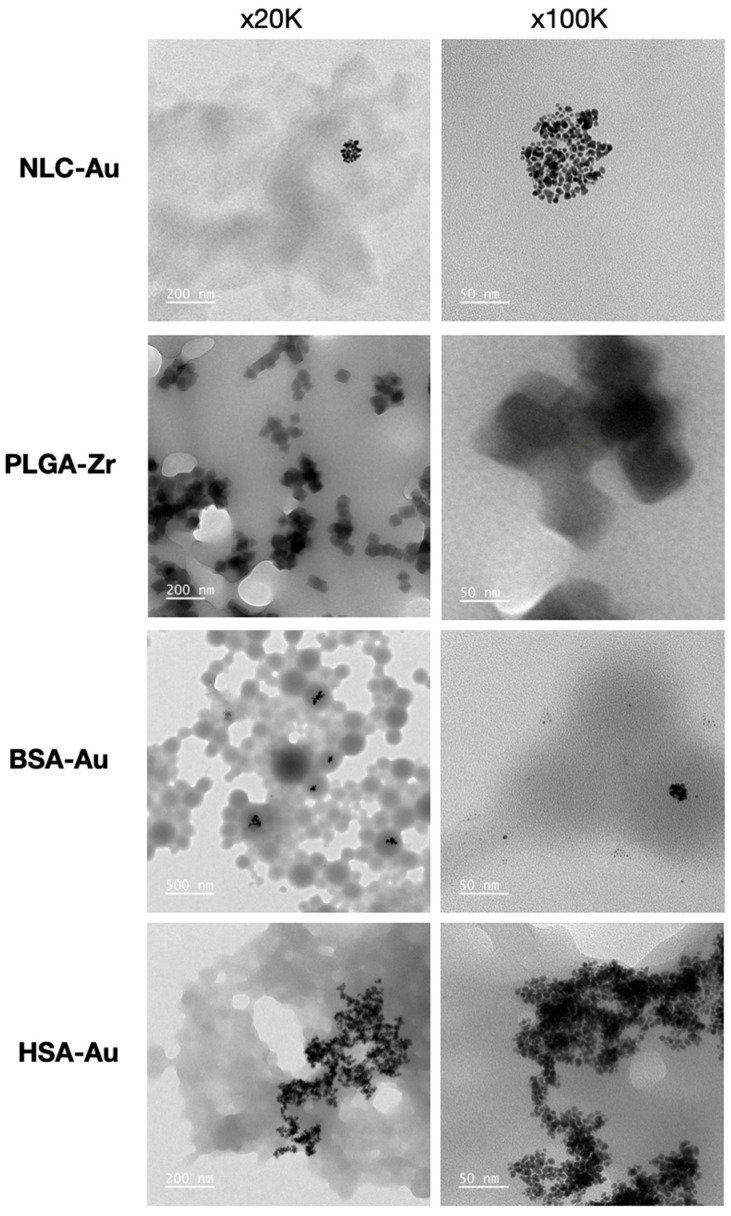
TEM micrographs of NP formulations.

The average particle size and PDI values of the Zr-MOF-labeled PLGA NPs were determined to be 327.2 nm and 0.235, respectively. For AuNP-labeled NLC NPs, the average particle size and PDI values were 414.3 nm and 0.307, respectively (Figure 1 and Figure 2). These results (Table 1) indicate that the NPs possess an appropriate distribution in the targeted biological systems and exhibit ideal characteristics for in vitro applications.

#### 2.1.2. Characterization of the Nanoparticles by FTIR

Having determined that the NPs show an appropriate size distribution, FTIR analysis was performed to determine the chemical structures of all the synthesized NPs for use in in vitro imaging studies.

**Figure 3:**
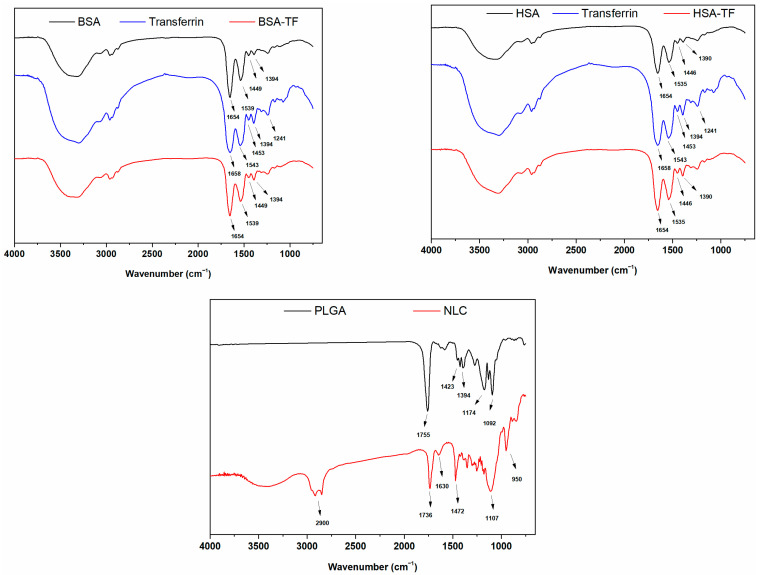
FTIR analysis results of the chemical structure of the synthesized NPs.

The characteristic FTIR peaks of BSA (Figure 3) were specified as follows: 1654 cm^−1^, related to C=O stretching vibrations; 1539 cm^−1^, attributed to amide II (N–H bending vibrations); 1449 cm^−1^, associated with amide III (C–N bending vibrations); and 1394 cm^−1^, explained by C–H stretching vibrations. Similarly, the FTIR spectrum of HSA (Figure 3) exhibits comparable peaks, which were classified as follows (cm^−1^): 1654 cm^−1^, corresponding to C=O stretching vibrations; 1535 cm^−1^, attributed to amide II (N–H bending vibrations); 1446 cm^−1^, associated with amide III (C–N bending vibrations); and 1390 cm^−1^, ascribed to C–H stretching vibrations [15].

The FTIR spectrum of Tf modification indicated the following peaks: 1658 cm^−1^, corresponding to C=O stretching vibrations of peptide bonds; 1543 cm^−1^, attributed to amide II band, N–H bending, and C–N stretching vibrations; 1453 cm^−1^, related to -CH_2_ and -CH_3_ bending vibrations; 1394 cm^−1^, assigned to CH_3_ symmetric bending vibrations; and 1241 cm^−1^, attributed to amide III, N–H bending, and C–N stretching vibrations. Tf modifications applied to BSA and HSA NPs result in minor changes observed at 1394 cm^−1^ and 1658 cm^−1^, indicating the successful integration of the Tf molecule into the NPs without structural degradation [27,28].

The characteristic FTIR peaks of the PLGA structure (Figure 3) were identified as follows: the peak at 1755 cm^−1^ corresponded to the C=O stretching vibrations of ester carbonyls; the peak at 1423 cm^−1^ was attributed to the -CH_3_ and -CH_2_ bending vibrations in the aliphatic structure of PLGA; the peak at 1174 cm^−1^ was assigned to the C=O stretching vibrations of ester bonds; and the peak at 1090 cm^−1^ was associated with C–O–C stretching vibrations [29].

Similarly, the characteristic FTIR peaks of the NLCs (Figure 3) were described as follows: the peak at 2900 cm^−1^ corresponded to aliphatic C–H stretching vibrations; the peak at 1736 cm^−1^ was attributed to the C=O stretching vibrations of carbonyl groups; the peak at 1630 cm^−1^ corresponded to the C=C stretching vibrations associated with unsaturated fatty acids derived from oleic acid; the peak at 1472 cm^−1^ was assigned to -CH_2_ and -CH_3_ bending vibrations; the peak at 1107 cm^−1^ was attributed to C–O–C stretching vibrations; and the peak at 950 cm^−1^ was related to the out-of-plane bending vibrations of C–H bonds linked to C=C double bonds [30].

According to the FTIR analysis results, all NPs were well synthesized, and Tf modification was successfully achieved.

### 2.2. In Vitro Studies

#### 2.2.1. Cytotoxicity of the NPs

**Figure 4:**
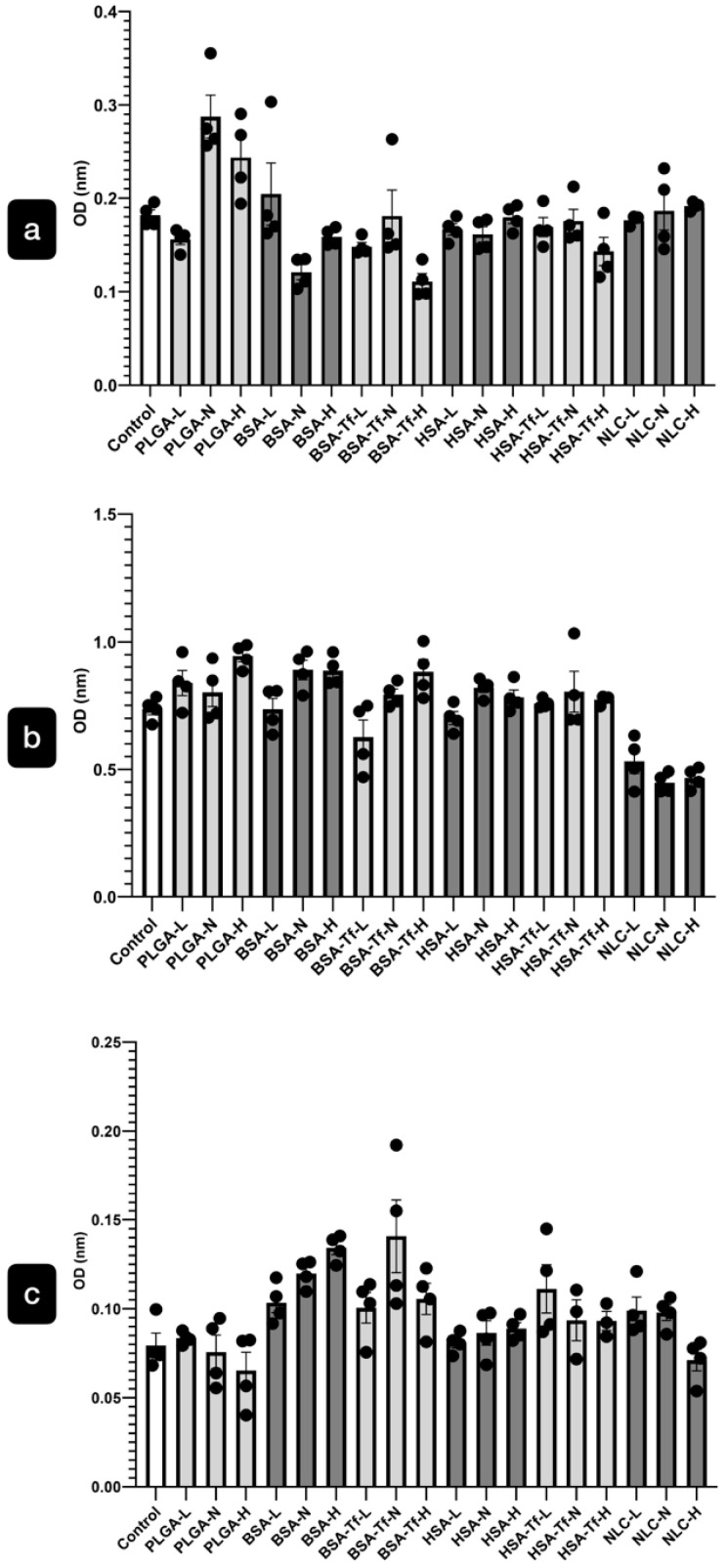
The cytotoxicity analysis of the NPs. Our results indicate that low (L: 15.62 µg/mL), normal (N: 31.25 µg/mL), and high (H: 62.5 µg/mL) doses of NP formulations including PLGA, BSA, BSA-Tf, HSA, HSA-Tf, and NLC did not cause any toxic effects on hBMECs (**a**), hBVPs (**b**), or hASTROs (**c**) compared to the control group after 3 h of incubation.

The cytotoxicity of the NPs was assessed using an LDH assay, a method for evaluating cell death. Our results indicated that none of the doses (15.62 µg/mL, 31.25 µg/mL, and 62.5 µg/mL) of PLGA, BSA, BSA-Tf, HSA, HSA-Tf, and NLC formulations exhibited any significant toxic effects on hBMECs (Figure 4a), hBVPs (Figure 4b), or hASTROs (Figure 4c) when compared to the control group after 3 h of incubation.

#### 2.2.2. NP Uptake

**Figure 5:**
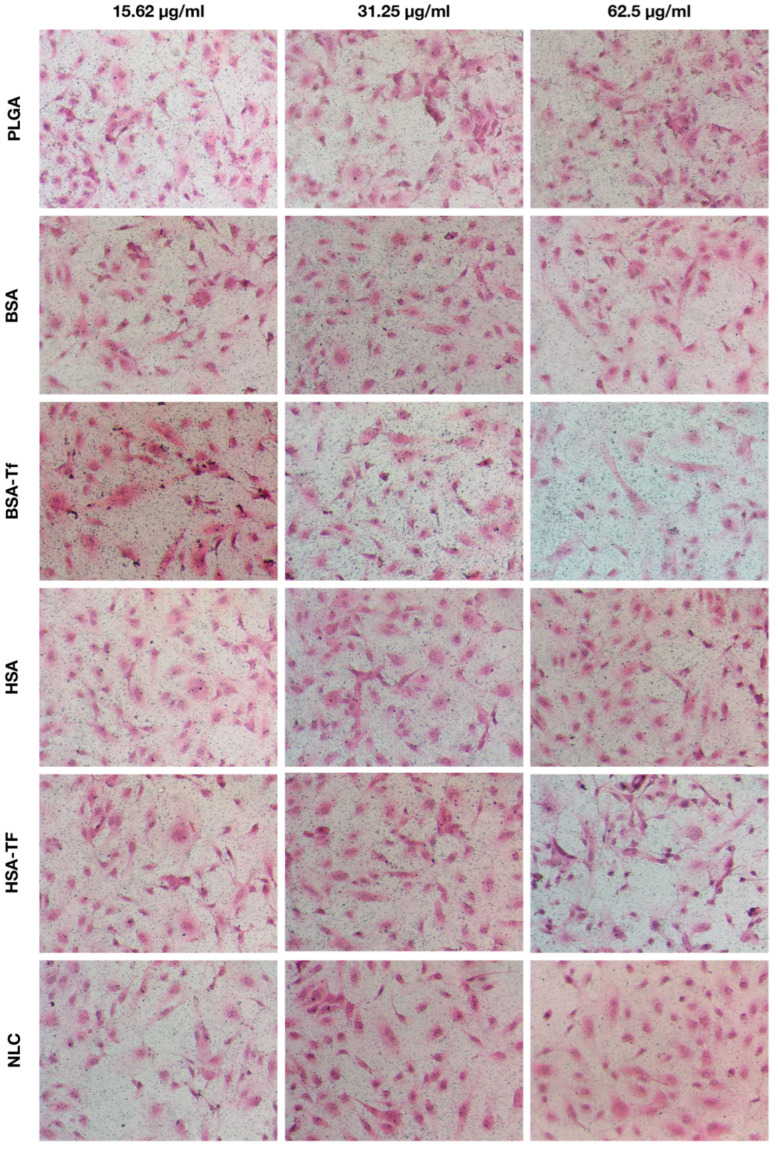
Representative micrographs showing the cellular uptake of different NPs according to the silver enhancer method (×400 maginification).

**Figure 6:**
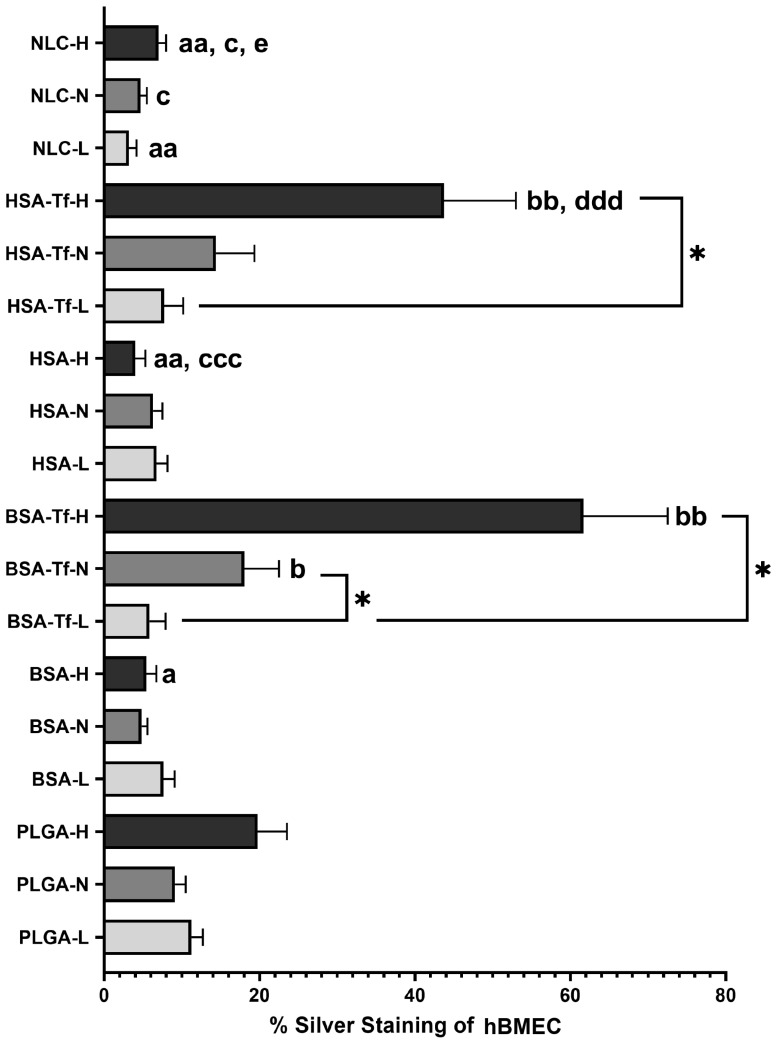
Percentage of cellular uptake of different NPs according to the silver enhancer method. * *p* < 0.05; ^a^ *p* < 0.05, ^aa^ *p* < 0.01 compared to the corresponding dose of %PLGA uptake; ^b^ *p* < 0.05, ^bb^ *p* < 0.01 compared to the corresponding dose of %BSA uptake; ^c^ *p* < 0.05, ^ccc^ *p* < 0.001 compared to the corresponding dose of %BSA-Tf uptake; ^ddd^ *p* < 0.001 compared to the corresponding dose of %HSA uptake; ^e^ *p* < 0.05 compared to the corresponding dose of %HSA-Tf uptake.

According to the silver enhancer method, the cellular uptake of PLGA in hBMECs was 11.2 ± 1.48% (*n* = 4), 9.11 ± 1.37% (*n* = 4), and 19.7 ± 3.77% (*n* = 4) for doses of 15.62 µg/mL, 31.25 µg/mL, and 62.5 µg/mL, respectively, with no statistically significant differences among these doses. Similarly, BSA exhibited uptake values of 7.62 ± 1.45% (*n* = 4), 4.81 ± 0.77% (*n* = 4), and 5.43 ± 1.28% (*n* = 4) for the same doses, while HSA showed values of 6.74 ± 1.41% (*n* = 4), 6.29 ± 1.21% (*n* = 4), and 4 ± 1.3% (*n* = 4), respectively, with no significant differences in cellular uptake among these doses. In contrast, BSA-Tf at 62.5 µg/mL and 31.25 µg/mL resulted in significantly higher cellular uptake compared to the 15.62 µg/mL dose, with uptake values of 61.7 ± 10.8% (*n* = 4), 18 ± 4.47% (*n* = 4), and 5.82 ± 2.09% (*n* = 4), respectively (*p* = 0.002 and *p* = 0.044, respectively). HSA-Tf at 62.5 µg/mL also showed significantly higher cellular uptake (43.7 ± 9.26%, *n* = 4) compared to the 15.62 µg/mL dose (7.71 ± 2.45%, *n* = 4) (*p* = 0.016). Interestingly, Tf conjugation to BSA (BSA-Tf) significantly increased the cellular uptake by hBMECs compared to the corresponding unconjugated forms (BSA) of NPs at doses of 62.5 µg/mL and 31.25 µg/mL (*p* = 0.001 and *p* = 0.021, respectively). Similar results were observed for HSA, where its Tf-conjugated form (HSA-Tf) showed significantly enhanced internalization by hBMECs at a dose of 62.5 µg/mL compared to the unconjugated form (HSA) (*p* < 0.001). NLC did not exhibit significant differences in cellular uptake across the different doses, with values of 3.2 ± 0.96% (*n* = 4), 4.66 ± 0.83% (*n* = 4), and 6.98 ± 0.99% (*n* = 4), for doses of 15.62 µg/mL, 31.25 µg/mL, and 62.5 µg/mL, respectively (Figure 5 and Figure 6). No significant differences in cellular uptake were observed for any NP formulation at different doses in hBVP and hASTRO cells (Figures S1–S4) (*p* = 0.16 and *p* = 0.097, respectively).

**Figure 7:**
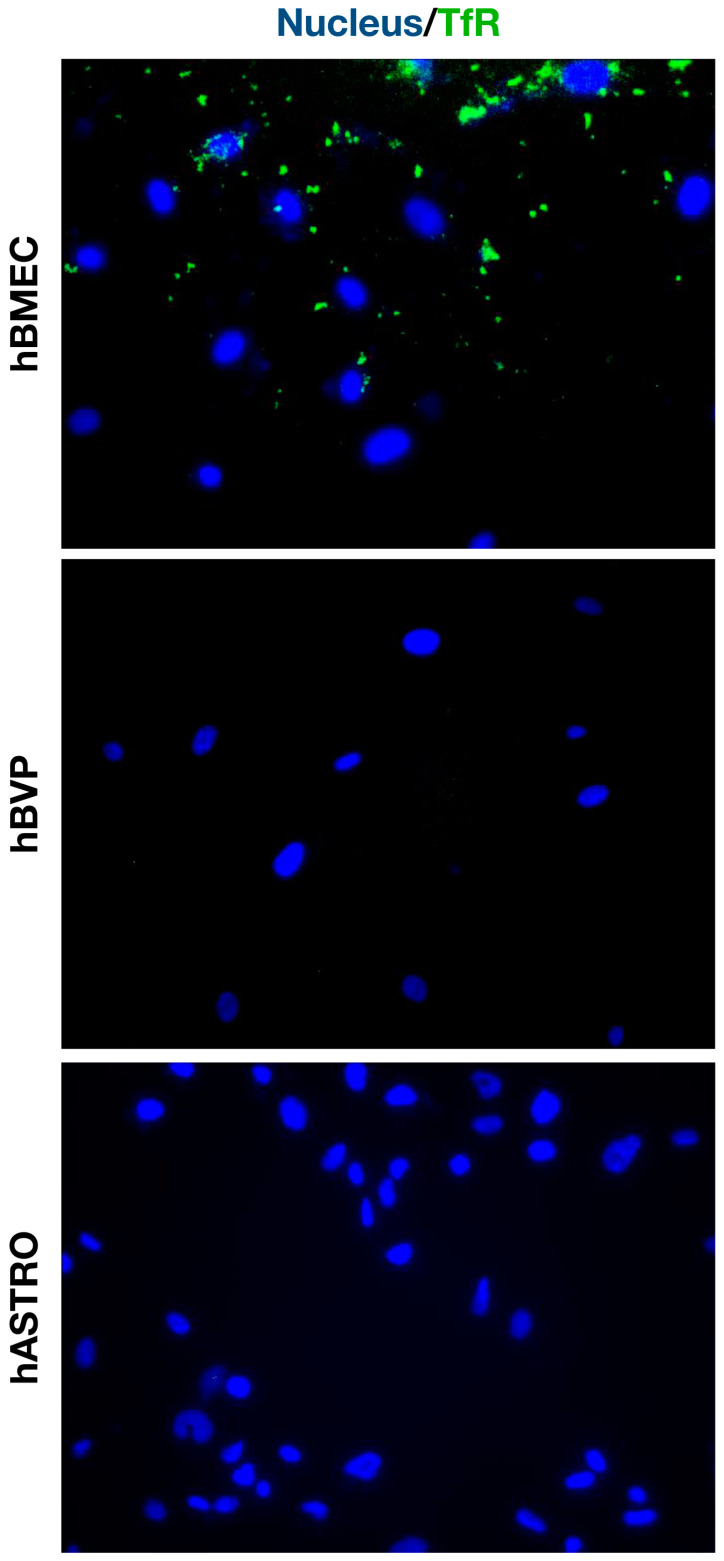
Micrographs demonstrating Tf receptor expression in hBMECs, while no Tf receptor expression was observed in hASTROs or hBVPs. TfR: transferrin receptor (×400 maginification).

To corroborate our observation that NP formulations with Tf ligands are significantly more taken up by hBMECs, we confirmed Tf receptor expression in these cells. In contrast, hBVP and hASTRO cells did not show any positivity for Tf receptors (Figure 7).

#### 2.2.3. Ultrastructural Features of hBMECs, hBVPs, and hASTROs

**Figure 8:**
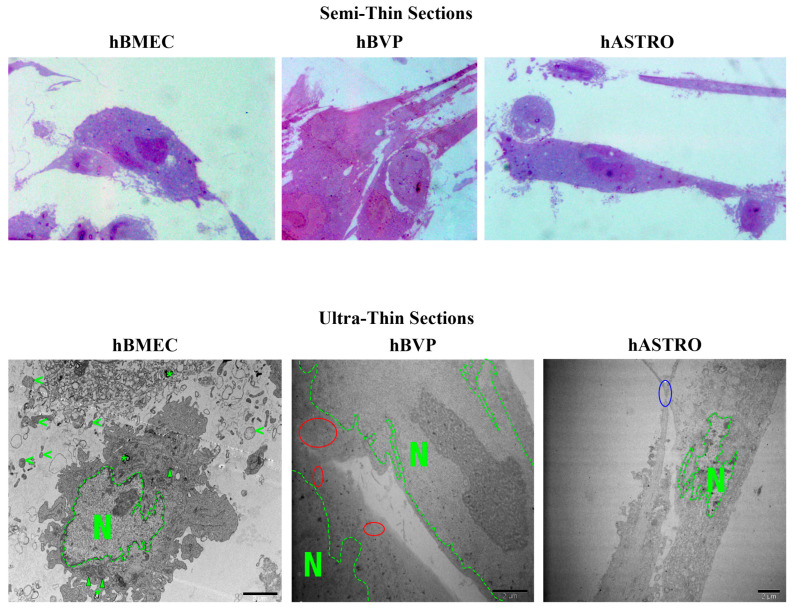
Semi-thin and ultra-thin sections of hBMECs, hBVPs, and hASTROs from control groups displaying healthy and active cellular profiles. N: nucleus, arrowhead: mitochondria, *: autophagic figures, <: extracellular vesicles, red cycles: small transport vesicles, blue cycle: cell-to-cell contact.

hBMECs exhibited a range of shapes and featured euchromatin-rich nuclei, usually containing more than one nucleolus. These nuclei often displayed indentations and folds. The mitochondria within hBMECs were elongated, and the cells were characterized by an abundance of free ribosomes, a prominent Golgi apparatus, and extensive rough endoplasmic reticulum (RER). Numerous caveolae were observed near the cell surfaces, and small transport vesicles were abundant in the cytoplasm, particularly at the periphery. Autophagic vacuoles, including laminar concentric myelin figures, were scattered throughout the cytoplasm. Various intercellular connections were evident. Thin and elongated cytoplasmic extensions were particularly noticeable at cell–cell contacts. Additionally, many extracellular vesicles were present, some of which were connected via cytoplasmic projections (Figure 8).

hBVPs typically feature one or two large, central nucleoli within euchromatin-rich, round or oval-shaped nuclei, occasionally showing nuclear indentations. The mitochondria are elongated and aligned parallel to the cell cytoplasm. Small transport vesicles are abundant throughout the cytoplasm, and various intercellular connections are evident. Autophagic vacuoles are also dispersed throughout the cytoplasm, often situated between larger vacuoles. Numerous multivesicular bodies are observed, and dilations of the rough endoplasmic reticulum (RER) are particularly prominent at the cytoplasmic periphery (Figure 8).

hASTROs display highly euchromatic, round, or oval nuclei with typically one central nucleolus. The mitochondria were elongated, and occasional vesicles were present in the cytoplasm. Some lipid droplets are identifiable. Various intercellular connections were also observed. Additionally, numerous vesicles showing different cargo products with varying electron densities, such as electron-lucent, electron-dense, and mixed types, were evident (Figure 8).

#### 2.2.4. Ultrastructural Analysis of Cellular Uptake for NP Formulations

**Figure 9:**
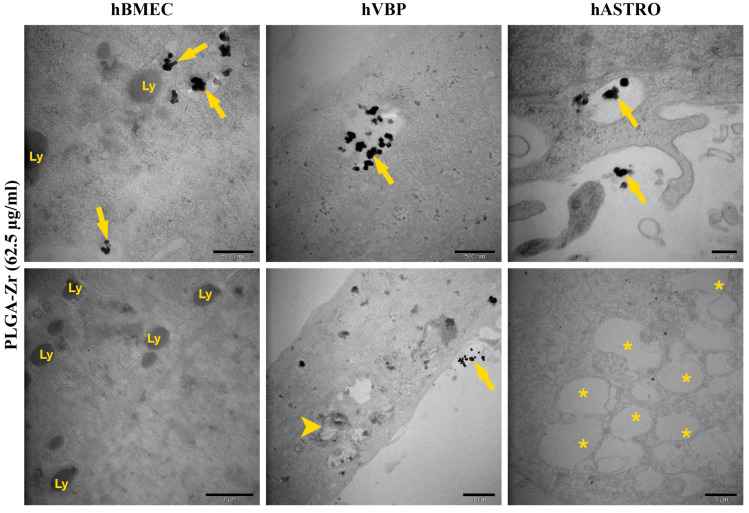
Representative TEM micrographs of PLGA application at 62.5 µg/mL in hBMECs, hBVPs, and hASTROs. PLGA, identified by its Zr marker (arrows), was observed within single-layered vesicles distributed throughout the cytoplasm of hBMECs, hBVPs, and hASTROs. Ly: lysosomes, arrowhead: large autophagic vacuoles, *: large vacuoles.

PLGA, identified by its Zr marker, was observed within single-layered vesicles distributed throughout the cytoplasm of hBMECs, hBVPs, and hASTROs. These vesicles typically contained multiple PLGA particles of varying sizes. Additionally, some PLGA particles were seen at the periphery of the vesicles, appearing to merge with or diffuse into the vesicle membranes. Many PLGA particles were also observed at the cell periphery, in contact with the surface membrane but not yet internalized, suggesting the initiation of NP uptake. No severe cellular injury was observed following PLGA application. However, an increase in lysosomes was evident in hBMECs, with the number of lysosomes correlating with the number of PLGA-containing vesicles. Furthermore, some of these vesicles were found in close proximity to, or in contact with, lysosomes. In contrast, PLGA application did not affect lysosomes in hBVPs or hASTROs. Nonetheless, large autophagic structures, which were absent in the control group, were occasionally observed in these cells. Additionally, some hASTROs contained large vacuoles accumulated in their cytoplasm (Table 2, Figure 9).

**Figure 10:**
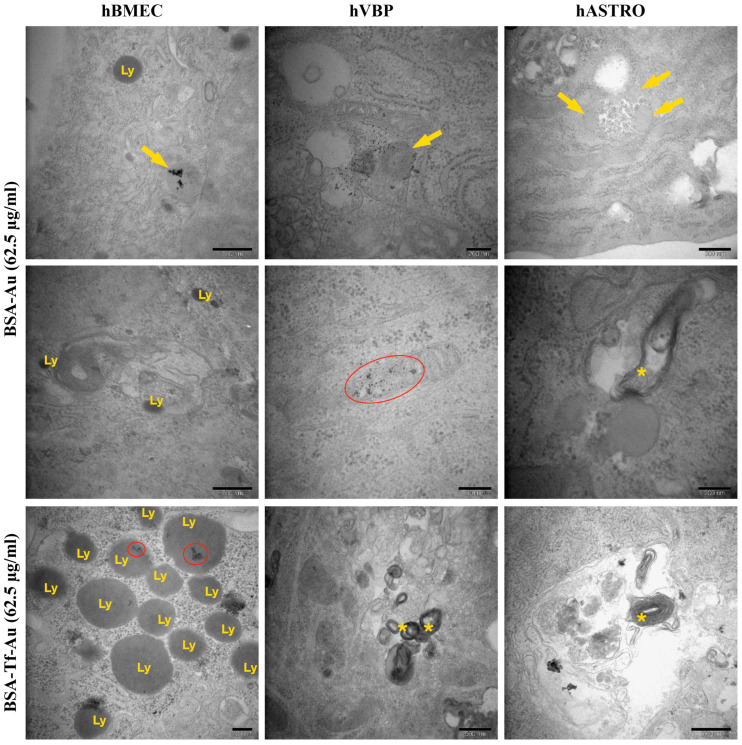
Representative TEM micrographs of BSA and BSA-Tf applications at 62.5 µg/mL in hBMECs, hBVPs, and hASTROs. BSA and BSA-Tf (arrows) were occasionally observed in hBMECs, hBVPs, and hASTROs. Ly: lysosomes, *: autophagic vacuoles, red cycles: Au particles accumulated in the lysosomes or in the mitochondria.

**Figure 11:**
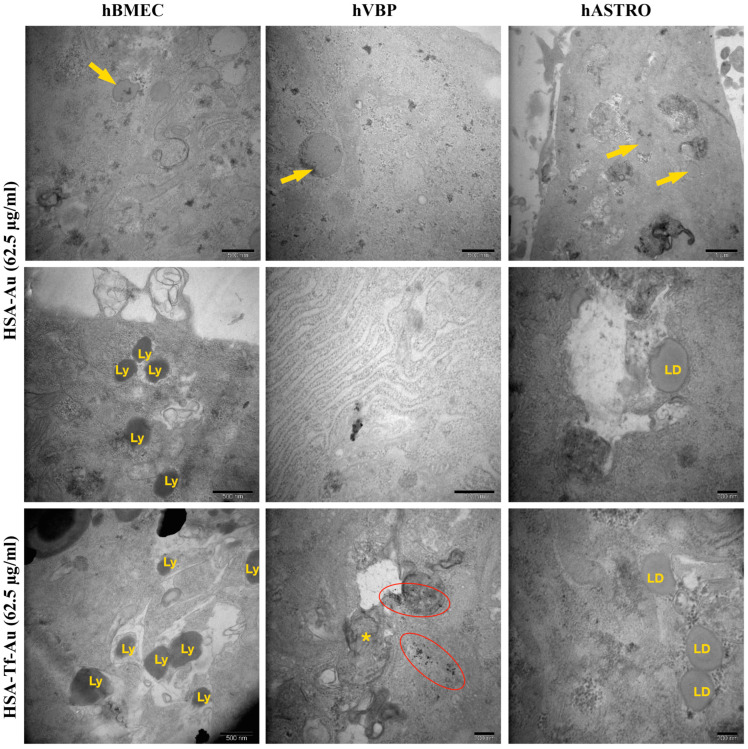
Representative TEM micrographs of HSA and HSA-Tf applications at 62.5 µg/mL in hBMECs, hBVPs, and hASTROs. HSA and HSA-Tf (arrows) were occasionally observed in hBMECs, hBVPs, and hASTROs. Ly: lysosomes, *: autophagic vacuoles, red cycles: Au particle accumulations in autophagic vacuoles, LD: lipid droplets.

BSA, BSA-Tf, HSA, and HSA-Tf were occasionally observed in hBMECs, hBVPs, and hASTROs. These NP formulations were typically located within the cytoplasm and were not associated with any vesicle-like structures. Additionally, Au particles, sometimes without BSA or HSA, were detected within the cytoplasm and occasionally within vesicles. Some Au particles accumulated in the lysosomes of hBMECs or in the mitochondria or autophagic vacuoles of hBVPs. While HSA-Tf and BSA-Tf showed similar distributions compared to their unconjugated forms in hBMECs and hBVPs, they were often found within large vesicles containing various cargo elements in hASTRO cells. Both BSA and HSA caused an increase in lysosomes in hBMECs. HSA-Tf and BSA-Tf also led to an increase in lysosomes, primarily located within large vesicles and autophagic vacuoles. In hBVPs, the application of BSA-Tf and HSA-Tf resulted in an increase in cellular debris within large autophagic vacuoles. In addition, some regions of hBVPs showed a significant dilation of the RER. Furthermore, HSA-Tf notably induced the formation of concentric myelin figures within the cytoplasm, along with numerous vesicles and large vacuoles containing Au particles alone in hBVPs and hASTROs. Applications of HSA and HSA-Tf led to the formation of many lipid droplets within the cytoplasm or vesicles, an increase in lysosomes, and occasionally the appearance of peroxisomes in all cell types (Table 2, Figure 10 and Figure 11).

**Figure 12:**
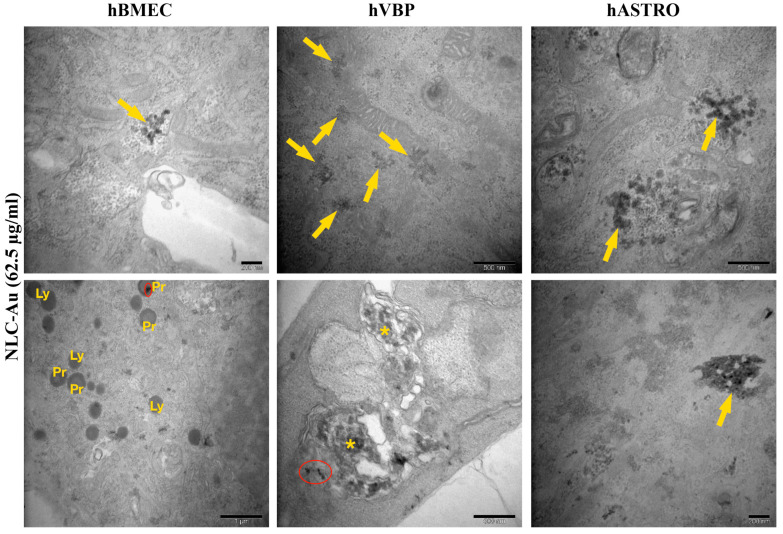
Representative TEM micrographs of NLC application at 62.5 µg/mL in hBMECs, hBVPs, and hASTROs. NLC (arrows) was occasionally observed in hBMECs, hBVPs, and hASTROs. Ly: lysosomes, Pr: peroxisomes, *: autophagic vacuoles, red cycles: Au particle accumulations in peroxisomes or autophagic vacuoles.

NLCs were observed throughout the cytoplasm of hBMECs, with some closely associated with lysosomes. Occasionally, Au particles were found alone within vesicles. Notably, in hBVPs, some NLCs appeared to merge with mitochondria. In hASTROs, NLCs were frequently located in large vesicles filled with various cargo elements. Additionally, in hBMECs, NLCs clearly induced an increase in the number of lysosomes and peroxisomes, with some Au particles accumulating in the peroxisomes. Conversely, NLC application in hBVPs led to an increase in large autophagic vacuoles containing cellular debris alongside Au particles. In contrast, no significant morphological changes were observed in hASTROs following NLC application (Table 1, Figure 12).

Furthermore, the mitochondria and other organelles showed no signs of injury from any of the NP applications in any of the sections observed. Additionally, none of the NP formulations were observed to enter the nuclei of hBMECs, hBVPs, or hASTROs.

## 3. Discussion

A wide range of drugs, including hydrophilic and hydrophobic small molecules, vaccines, and biological macromolecules, can be delivered using nanoparticles (NPs). The key objectives when designing NPs as drug delivery systems are to manage the particle size, surface characteristics, and the release profile of the active agents, ensuring site-specific drug action at an optimal therapeutic rate and dose. For NPs to be effective in drug delivery, they must meet several criteria, including biocompatibility, drug compatibility, appropriate biodegradation kinetics, and suitable mechanical properties. Additionally, NP delivery systems are particularly advantageous in cancer treatment, as they enhance the accumulation of anticancer agents in tumor cells while minimizing exposure to healthy tissues [31].

Polymer-based NPs are submicron-sized colloidal particles in which therapeutic agents can be encapsulated within the polymeric matrix or conjugated to the surface. These NPs serve as effective carriers for delivering different types of biomolecules to specific sites in vivo. The copolymer poly(lactic-*co*-glycolic acid) (PLGA) has been widely used due to its excellent biocompatibility and biodegradability. PLGA, one of the most commonly used biodegradable polymers in nanomedicine, undergoes hydrolysis into lactic and glycolic acid, which are metabolized via the Krebs cycle and excreted as carbon dioxide and water, resulting in minimal systemic toxicity. Both the U.S. Food and Drug Administration (FDA) and the European Medicines Agency (EMA) have approved PLGA for use in various drug delivery systems [31,32,33]. According to the DLS results, the particle sizes of our synthesized PLGAs were analyzed as 327.2 ± 35.3 nm, and the PDI value was 0.235 ± 0.016. The particle sizes were confirmed by TEM analyses, and the surface morphology of the nanoparticle was determined as spherical. The particle sizes and surface morphology of the synthesized PLGA NP show properties very similar to those of the synthesized PLGA structures in the literature in many respects [34,35,36].

Among albumins, bovine serum albumin (BSA) and human serum albumin (HSA) are widely studied for drug delivery and have gained significant attention in cancer diagnosis and therapy. BSA is particularly promising for cancer therapeutics due to its numerous advantages. HSA, which shares 75.6% sequence homology with BSA, has a similar structure and is also capable of binding most drugs. Both BSA and HSA are naturally derived, non-toxic, non-immunogenic, biocompatible, and biodegradable, making their materials ideal for drug delivery. Their molecular structures feature multiple drug-binding sites, allowing the encapsulation of substantial amounts of bioactive compounds or drugs. This high binding capacity makes these albumins effective and well-tolerated delivery vehicles, capable of transporting various therapeutics without causing significant side effects. Moreover, their well-defined protein structures, rich in charged bioactive amino acids, facilitate covalent bonding and electrostatic adsorption with positively or negatively charged biomolecules [37,38,39,40]. However, it should also be noted that although human exposure to BSA is common via both dietary and medicinal routes, pharmaceuticals take steps to limit BSA exposure and reduce any potential risk of bovine spongiform encephalopathy. And while immune responses are not typically reported, it should be noted that one study found anti-BSA antibodies in 55% of 60 healthy blood donors [41]. The particle sizes of BSA and HSA NPs prepared by the desolvation technique were analyzed as 223.3 ± 15.8 nm and 114.5 ± 7.5 according to the DLS results, and the PDI values were found to be 0.189 ± 0.020 and 0.228 ± 0.018, respectively. TEM analyses confirmed the particle sizes and morphology of the NPs, which were determined to be spherical. FTIR analyses confirmed the structural composition of NPs. After Tf modification, the particle sizes of the synthesized BSA and HSA NPs reached 364.0 ± 22.1 nm and 181.3 ± 14.2 nm, respectively, and the PDI values reached 0.324 ± 0.045 and 0.352 ± 0.033, respectively. Both synthesized and Tf-modified NPs exhibited properties similar to their equivalents in the literature [42,43].

Lipid-based NPs encompass a variety of structural subsets and offer numerous advantages as drug delivery systems as well, including formulation simplicity, self-assembly, biocompatibility, high bioavailability, and the ability to carry large payloads. Their physicochemical properties can also be tailored to modulate biological behavior, making them the most common class of FDA-approved nanomedicines. Nanostructured lipid carriers have emerged as a novel drug delivery system, consisting of a nano-sized colloidal mixture of solid and liquid lipids in their core. This design enhances drug solubility and permeability, making these carriers ideal for delivering drugs through challenging routes. Despite these advantages, potential cytotoxicity and the risk of irritation due to surfactants remain significant drawbacks [24,26]. Our DLS analysis results for the synthesized NLC NPs determined the particle size as 414.3 ± 28.6 nm and the PDI value as 0.307 ± 0.061. The particle sizes of NLCs, which have relatively larger particles compared to other synthesized NP groups, were confirmed by TEM analysis, and their chemical structures were characterized by FTIR analysis [44,45].

Our in vitro results showed that a 3 h exposure to NPs, including PLGA, BSA, BSA-Tf, HSA, HSA-Tf, and NLC, did not cause any cytotoxicity in human microvascular endothelial cells (hBMECs), primary human brain vascular pericytes (hBVPs), or primary human astrocytes (hASTROs) at doses of 15.62 µg/mL, 31.25 µg/mL, and 62.5 µg/mL. Subsequent analysis confirmed that all cell types internalized all NP formulations at each dose.

Modifying the surface of NPs with targeting ligands enables specific interactions with cell surface receptors, enhancing internalization, which is a concept in nanomedicine known as active targeting. Common targeting ligands include peptides, small molecules, proteins, antibodies, antibody fragments, and nucleic acids [26]. Among these, transferrin (Tf) is frequently used in brain-related studies [46,47], including one of our previous investigations [15]. In this study, we used Tf as a ligand for our albumin-based NPs to target endothelial cells and assess whether these NPs could facilitate more specific interactions, potentially aiding in the crossing of the human BBB. Notably, BSA-Tf and HSA-Tf exhibited significantly higher, dose-dependent internalization in hBMECs compared to BSA and HSA alone, but this effect was not observed in hBVPs or hASTROs. These findings demonstrate the potential of targeting NPs with specific ligands, such as Tf, as only hBMECs express Tf receptors among these three cell types.

Our ultrastructural analysis revealed that hBMECs, hBVPs, and hASTROs generally displayed healthy and active cellular profiles, with cell-specific structural features. However, when evaluating the interaction of NP formulations with the cells, notable differences were observed depending on the specific NP formulation and cell type.

In hBMECs, PLGA particles were primarily found in single-layered vesicles, indicating that the uptake mechanism likely involved endocytosis, a process consistent with previous studies [26,48,49]. Notably, the increase in lysosomal numbers in hBMECs following PLGA exposure suggests that these NPs are processed by the endosomal–lysosomal pathway, a critical aspect of cellular response to foreign particles [50,51]. This lysosomal involvement could indicate a degradation or recycling mechanism as part of the cellular handling of PLGA NPs. However, the lack of significant morphological damage suggests that PLGA NPs are well tolerated by endothelial cells at the concentrations tested. In hBVPs and hASTROs, PLGA NPs were also observed in single-layered vesicles, yet without the pronounced increase in lysosomes seen in hBMECs. The presence of large autophagic structures in hBVPs and hASTROs suggests that PLGA NPs may activate autophagy in these cells, potentially as a protective mechanism to clear intracellular particles [52].

BSA and HSA NPs, labeled with gold NPs (AuNPs), displayed distinct uptake profiles in the different cell types. Albumin NPs are known for their ability to enhance the circulation time of drugs and their preferential uptake by certain cell types due to albumin receptor interactions [53]. In hBMECs, BSA and HSA NPs were internalized but not associated with vesicular structures, suggesting passive diffusion or direct cellular uptake. The observed increase in lysosomal activity in hBMECs following BSA and HSA exposure suggests that these NPs may be routed through the lysosomal degradation pathway, though without causing cytotoxicity. Interestingly, in hBVPs and hASTROs, BSA and HSA NPs were observed within the cytoplasm without vesicular association, and AuNPs were found to accumulate in mitochondria and autophagic vacuoles. This mitochondrial accumulation may indicate potential oxidative stress responses, as some of these NPs are known to induce mitochondrial dysfunction [54,55,56]. Additionally, the formation of autophagic vacuoles in these cells points to autophagy as a key response mechanism, likely involved in the clearance of foreign NPs [52,57]. This is a critical finding, as it suggests that BSA and HSA NPs could induce mild cellular stress in pericytes and astrocytes, which may influence their long-term use in therapeutic delivery to the brain. While no enhancement of cell death was observed in the present study, more sensitive assays of cell stress, redox state, and cellular dysfunction are therefore required to confirm that these NP formulations are well tolerated.

AuNP-labeled NLCs showed modest uptake across all cell types, with no significant differences between doses. In hBMECs, NLCs were frequently associated with lysosomes and peroxisomes, suggesting that these NPs are processed by cellular detoxification and degradation pathways. The accumulation of Au particles in peroxisomes may point to a mild oxidative stress response, as peroxisomes are involved in reactive oxygen species (ROS) metabolism [58]. In hBVPs, the presence of large autophagic vacuoles suggests that NLCs may trigger autophagy, possibly as a protective mechanism against NP-induced stress similar to albumin-based NPs. In hASTROs, NLCs were frequently found within large vesicles containing various cargo elements, indicating that astrocytes may sequester NLCs in vesicular compartments to minimize intracellular exposure. This suggests that astrocytes, as supportive cells in the BBB, may play a protective role in preventing the accumulation of NPs in the CNS, a process that could be exploited for targeted drug delivery.

Successful transcytosis requires NPs to resist degradation and be directed through the cell for exocytosis on the opposite side of the BBB [20]. As a limitation of our study, we acknowledge that while our TEM analyses confirm cellular internalization, they do not verify complete transcytotic passage across the cell. Future studies could address this by incorporating additional assays to monitor the entire intracellular journey of the NPs, such as advanced TEM techniques or transwell models that track NP transport through cells and across the endothelial layer. This limitation highlights the need for further investigation into endosomal escape and intracellular routing mechanisms to confirm full transcytosis potential. Thus, additional studies are essential to determine whether these NPs can fully traverse the BBB and reach the CNS.

## 4. Materials and Methods

### 4.1. Synthesis of Markers (Gold Nanoparticles and Zr-Based Metal–Organic Frameworks) for In Vitro Imaging Studies

In order to facilitate an easy and effective imaging of PLGA, BSA, HSA, and NLC NPs used in in vitro studies, firstly, two nano-sized markers (gold NPs (AuNPs) and zirconium-based metal–organic frameworks (Zr-MOFs)) were synthesized and used in labeling studies.

AuNPs used for imaging were synthesized using the Brust–Schiffrin method, as established in the previous literature [59], to achieve small particle sizes and narrow size distribution. In this process, HAuCl_4_ (Cat# 520918, Sigma-Aldrich, Burlington, MA, USA) (0.1 mM, 1 equivalent) was reduced using NaBH_4_ (Cat# 213462, Sigma-Aldrich, Burlington, MA, USA) (1 M, 20 equivalents) in the presence of a GSH (Cat# G4251, Sigma-Aldrich, Burlington, MA, USA) ligand. After the synthesis, the mixture was purified with methanol and centrifuged at 15,000 rpm for 15 min; then, AuNPs were lyophilized.

The synthesis of zirconium (Zr)-based metal–organic frameworks (MOFs) was performed via the solvothermal method. Initially, 0.3 mmol ZrCl_4_ (Cat# 221880, Sigma-Aldrich, Burlington, MA, USA) was dissolved in 10 mL of dimethylformamide (DMF) and formic acid (9:1, *v*:*v*) solution, and simultaneously, 0.3 mmol terephthalic acid (Cat# 100-21-0, Merck KGaA, Darmstadt, Germany) was dissolved in 10 mL of DMF. Both solutions were combined in a glass reactor and stirred for 1 h at room temperature. The mixture was then incubated at 120 °C for 24 h without stirring. The resulting MOF structures were separated by centrifugation at 15,000 rpm for 15 min. A solvent exchange process with methanol was applied to remove DMF from the MOF structures completely. The obtained MOF structures were dried at 120 °C for 24 h and activated at 150 °C for 24 h.

Both synthesized markers, AuNPs and Zr-MOFs, were stored at +4 °C until they were used to synthesize labeled NPs (PLGA, BSA, HSA, and NLC) employed in cell studies.

### 4.2. Synthesis of BSA and HSA Nanoparticles

The synthesis of marker-loaded BSA and HSA NPs was carried out using the desolvation method. Specifically, AuNP solution (0.5 mL, 5 mg/mL) was added to 1 mL of BSA (Cat#A2153, Sigma-Aldrich, Burlington, MA, USA) or HSA (Cat#A1653, Sigma-Aldrich, Burlington, MA, USA) solution (30 mg/mL), and the pH was adjusted to 9. The prepared solution was stirred at 1200 rpm for 30 min. During nanoparticulation, 2 mL of acetone (1 mL/min) was added dropwise, followed by 8 µL of 25% glutaraldehyde (Cat# 820603, Merck KGaA, Darmstadt, Germany) solution to the reaction mixture. After gentle mixing for an hour, the mixture was centrifuged at 15,000 rpm for 20 min. The synthesized products were purified twice with distilled water, lyophilized for in vitro studies and Tf conjugation, and stored at +4 °C.

### 4.3. Transferrin Conjugation of HSA and BSA Nanoparticles

Tf conjugation to AuNP-labeled NPs was performed using EDC/NHS chemistry. Briefly, 1 mL of AuNP-loaded BSA and HSA NPs (20 mg/mL) was treated with 0.25 mL of 1-ethyl-3-(3-dimethylaminopropyl)carbodiimide (EDC) (Cat# E7750, Sigma-Aldrich, Burlington, MA, USA) and *N*-hydroxysulfosuccinimide (NHS) (Cat# 804518, Merck KGaA, Darmstadt, Germany) solutions (0.5 mg/mL). Following this step, 1 mL of Tf (Cat# T8158, Sigma-Aldrich, Burlington, MA, USA) solution (5 mg/mL) was added, and the mixture was stirred at room temperature for 2 h. The solution was centrifuged and purified twice with distilled water, and the synthesized Tf-conjugated BSA and HSA NPs were lyophilized and stored at +4 °C for in vitro studies. Tf-conjugated NPs were expressed as BSA-Tf or HSA-Tf.

### 4.4. Synthesis of NLCs

The NLCs were synthesized using the hot homogenization method. A solid–liquid lipid mixture (150 mg Compritol ATO) (Cat #77538-19-3, Gattefosse, Saint-Priest, France): Oleic Acid (Cat# E7750, Sigma-Aldrich, Burlington, MA, USA), (1:1 *w*:*w*) along with 5 mg of AuNPs was heated to 80 °C to ensure homogeneous mixing. A 10 mL 1.5% Tween 80 solution was heated to the same temperature and added to the lipid phase, followed by homogenization at 20,000 rpm for 5 min. After homogenization, 10 mL of cold distilled water was added to form the solid lipid matrix. The solution was centrifuged at 15,000 rpm for 20 min and purified twice with distilled water. The marker-loaded NLCs were lyophilized and stored at +4 °C for in vitro studies.

### 4.5. Synthesis of PLGA Nanoparticles

For the synthesis of PLGA NPs, a PLGA solution (25 mg/mL in DMSO) was prepared and mixed with 10 mg of activated Zr-MOF at room temperature for 1 h. The organic phase was gradually added to 20 mL of an aqueous solution containing 1% polyvinyl alcohol (PVA) at a controlled flow rate of 0.5 mL/min. The mixture was subjected to ultrasonic treatment at 30% amplitude for 5 min using a Bandelin Sonoplus MS73 probe (Berlin, Germany). After encapsulation, the PLGA NPs were centrifuged at 15,000 rpm for 20 min and purified twice in ethanol. The purified NPs were lyophilized and stored at +4 °C for in vitro studies.

### 4.6. Cell Culture

Primary human microvascular endothelial cells (hBMECs) (Cat# cAP-0002, Angio-Proteomie, Boston, MA, USA), primary human brain vascular pericytes (hBVPs) (Cat# SC-1200, Caltag Medsystems, Buckingham, UK), and primary human astrocytes (hASTROs) (Cat# SC-1800, Caltag Medsystems, Buckingham, UK) were cultured in their respective growth media: endothelial cell growth medium-2 (EGM-2; Cat# CC-3162, Lonza Group, CH, Basel, Switzerland), pericyte growth medium (Cat# SC-1201, Caltag Medsystems, UK), and astrocyte medium (Cat# 1801, ScienceCell Research Laboratories, Carlsbad, CA, USA). After cultivation and proliferation, the cells were trypsinized and centrifuged. A total of 3.5 × 10^4^ and 7 × 10^3^ cells were seeded into 6-well plates, with or without glass coverslips, and into 96-well plates, using 1000 µL and 100 µL of media per well, respectively. The cells were then allowed to reach confluence before proceeding with subsequent experimental procedures.

### 4.7. In Vitro Application of Nanoparticles

In selecting the doses for this study, we first reviewed the doses used for NPs in brain transport studies across various hypotheses in the literature. According to these sources, the range of doses studied for NPs such as BSA, HSA, PLGA, and NLC covers a broad spectrum (1 μg/mL to 1000 μg/mL). Based on this information, we selected common doses that could comprehensively represent each nanoparticle type in our study, allowing for comparative evaluation of their transport efficiencies [60,61,62,63,64,65,66]. Therefore, each NP formulation including PLGA, BSA, BSA-Tf, HSA, HSA-Tf, and NLC was applied at low (15.62 μg/mL), normal (31.25 μg/mL), and high (62.5 μg/mL) concentrations, while the control group received no NP treatment.

After 3 h of NP exposure, the media were collected and stored at −80 °C. The cells were then fixed with either 4% paraformaldehyde (PFA) for light microscopy or 2.5% glutaraldehyde + 4% PFA in 0.1 M PIPES buffer at pH 7.2 for TEM analysis.

### 4.8. Cytotoxic Assay

The collected media from the experimental groups were used to measure the release of lactate dehydrogenase (LDH), an indicator of cytotoxicity, using the CytoTox kit (Cat# G1780, Promega Corporation, Madison, WI, USA) according to the manufacturer’s instructions.

### 4.9. Histological Analysis

To identify Tf receptor expression, the fixed cells were washed with PBS and then permeabilized. Subsequently, they were blocked with 1% BSA for 30 min. The cells were then incubated overnight at 4 °C with primary mouse anti-Tf receptor antibody (diluted 1:500, Cat# G1/221/12, Developmental Studies Hybridoma Bank, Iowa City, IA, USA). After washing with PBS, the cells were incubated with donkey anti-mouse IgG H&L, (Alexa Fluor 488, Cat# ab150109, Abcam, Cambridge, UK). Finally, counterstaining was performed using Hoechst dye (Cat# 62249, ThermoFisher Scientific, Waltham, MA, USA).

Since the NP formulations were labeled with either Zr or Au and their sizes were undetectable under light microscopy, a silver enhancer kit (Cat# SE100, Merck KGaA, Darmstadt, Germany) was used to amplify the NPs’ size for identification in hBMECs, hBVPs, and hASTROs under light microscopy, as previously reported [67,68]. Firstly, the fixed cells were washed with distilled water. Secondly, they were then covered with the silver enhancer mixture and observed under light microscopy to monitor the reaction. Once the NPs became visible and the background was acceptable, the cells were rinsed with distilled water to halt the reaction. Next, the cells were fixed with 2.5% sodium thiosulfate solution and washed again. Lastly, hematoxylin and eosin staining was applied as a counterstain. The stained areas of the cells, induced by the silver enhancer, were quantified using ImageJ software (v1.51) [69]. Initially, micrographs were separated into color channels using the color deconvolution tool. The subsequent step involved measuring the area of the NPs with the threshold tool. Following this, the total cell area was assessed in the eosin channel, also using the threshold tool. Finally, the NP area was divided by the total cell area to calculate the percentage of NP staining induced by silver enhancer in hBMECs, hBVPs, and hASTROs.

For ultrastructural analysis, only the high dose (62.5 µg/mL) of NP applications was used. The fixed cells on coverslips were treated with 1% osmium tetroxide and 1.5% potassium ferrocyanide in 0.1 M PIPES buffer as a secondary fixative. The samples were then washed with distilled water and incubated overnight at 4 °C in 0.5% uranyl acetate for negative staining. After rinsing, the cells were dehydrated through an ascending ethanol series, and embedding was performed using the Agar 100 premix kit (AGR1140, Agar Scientific, Rotherham, UK). The coverslips were inverted onto embedding capsules filled with resin, and the samples were polymerized at 60 °C for 48 h. The coverslips were subsequently removed from the resin blocks to yield a monolayer of cells on the surface. Semi-thin sections (300–500 nm) were obtained and stained with toluidine blue, while ultra-thin sections (70–90 nm) were prepared using an ultramicrotome (Leica EM UC7, Wetzlar, Germany) and mounted on copper grids. Finally, lead citrate was applied for post-staining. The samples were then examined under a TEM (JEOL 1400 or JEM-1011, Tokyo, Japan).

## 5. Conclusions

This study provides a comprehensive comparison of various NP formulations, including PLGA, BSA, HSA, and NLCs, in terms of their cellular uptake and intracellular interactions with key cell types of the BBB including hBMECs, hBVPs, and hASTROs. Our findings demonstrate that Tf-conjugated NPs, specifically BSA-Tf and HSA-Tf, significantly enhance uptake in hBMECs, highlighting the potential of ligand-targeted NPs to selectively engage with BBB endothelial cells. Additionally, our ultrastructural results reveal distinct cellular processing pathways for different NP formulations across BBB-associated cell types. Moreover, the autophagic process appears to be a crucial mechanism for NP handling in pericytes and astrocytes. While none of the NP formulations induced acute cytotoxicity, the activation of lysosomal and autophagic pathways suggests that long-term exposure and potential cellular stress responses warrant further investigation.

## Figures and Tables

**Table 1:** Particle size and PDI values of synthesized NPs according to DLS analysis.

NP	Particle Size (nm)	PDI
PLGA	327.2 ± 35.3	0.235 ± 0.016
BSA	223.3 ± 15.8	0.189 ± 0.020
BSA-Tf	364.0 ± 22.1	0.324 ± 0.045
HSA	114.5 ± 7.5	0.228 ± 0.018
HSA-Tf	181.3 ± 14.2	0.352 ± 0.033
NLC	414.3 ± 28.6	0.307 ± 0.061

**Table 2:** Ultrastructural observations of the application of PLGA, BSA, BSA-Tf, HSA, HSA-Tf, and NLC NP formulations on hBMECs, hBVPs, and hASTROs at 62.5 µg/mL doses after 3 h of exposure.

	Cell	hBMEC	hBVP	hASTRO
NP				
PLGA		NPs were found in single-layered vesicles. NP-containing vesicles were in contact with the lysosomes. Increase in the number of lysosomes.	NPs were found in single-layered vesicles. Presence of large autophagic structures.	NPs were found in single-layered vesicles. Presence of large autophagic structures. Presence of large vacuoles within the cytoplasm.
BSA		NPs were observed in the cytoplasm without being associated with vesicle-like structures. Increase in the number of lysosomes.	NPs were observed in the cytoplasm without being associated with vesicle-like structures. Au particles accumulated in the mitochondria or autophagic vacuoles.	NPs were observed in the cytoplasm without being associated with vesicle-like structures. Au particles accumulated in the mitochondria or autophagic vacuoles.
BSA-Tf		NPs were observed in the cytoplasm without being associated with vesicle-like structures. Increase in the number of lysosomes.	NPs were observed in the cytoplasm without being associated with vesicle-like structures. Au particles accumulated in the mitochondria or autophagic vacuoles. There was an increase in cellular debris. Certain regions showed significant dilation of the RER.	NPs were observed in the cytoplasm without being associated with vesicle-like structures. Au particles accumulated in the mitochondria or autophagic vacuoles. NPs were frequently found within large vesicles.
HSA		NPs were observed in the cytoplasm without being associated with vesicle-like structures. Increase in the number of lysosomes, lipid droplets, and vesicles. Increase in the number of peroxisomes.	NPs were observed in the cytoplasm without being associated with vesicle-like structures. Au particles accumulated in the mitochondria or autophagic vacuoles. Increase in the number of lysosomes, lipid droplets, and vesicles. Increase in the number of peroxisomes.	NPs were observed in the cytoplasm without being associated with vesicle-like structures. Au particles accumulated in the mitochondria or autophagic vacuoles. Increase in the number of lysosomes, lipid droplets, and vesicles. Increase in the number of peroxisomes.
HSA-Tf		NPs were observed in the cytoplasm without being associated with vesicle-like structures. Increase in the number of lysosomes, lipid droplets, and vesicles. Increase in the number of peroxisomes.	NPs were observed in the cytoplasm without being associated with vesicle-like structures. Au particles accumulated in the mitochondria or autophagic vacuoles. There was an increase in cellular debris. Certain regions showed significant dilation of the RER. Formation of concentric myelin figures within the cytoplasm were induced. Increase in the number of lysosomes, lipid droplets, and vesicles. Increase in the number of peroxisomes.	NPs were observed in the cytoplasm without being associated with vesicle-like structures. Au particles accumulated in the mitochondria or autophagic vacuoles. NPs were frequently found within large vesicles. Increase in the number of lysosomes, lipid droplets, and vesicles. Increase in the number of peroxisomes.
NLC		NPs were found within the cytoplasm without association with vesicle-like structures. Increase in the number of lysosomes and peroxisomes. Some Au particles accumulated in the peroxisomes.	NPs were found within the cytoplasm without association with vesicle-like structures. NPs appeared to merge randomly with the mitochondria. An increase in large autophagic vacuoles containing cellular debris and Au particles.	NPs were found within the cytoplasm without association with vesicle-like structures. NPs were frequently found within large vesicles.

## Data Availability

Dataset available on request from the authors.

## Supplementary Materials

The following supporting information can be downloaded at: https://www.mdpi.com/article/10.3390/ph17121567/s1, Figure S1: representative micrographs showing the cellular uptake of different NPs according to the silver enhancer method for hBVPs; Figure S2: percentage of cellular uptake of different NPs according to the silver enhancer method for hBVPs (*n* = 4); Figure S3: representative micrographs showing the cellular uptake of different NPs according to the silver enhancer method for hASTROs; Figure S4: percentage of cellular uptake of different NPs according to the silver enhancer method for hASTROs (*n* = 4).
