# Supplementary figures and images for: Comparison of Drug Delivery Systems with Different Types of Nanoparticles in Terms of Cellular Uptake and Responses in Human Endothelial Cells, Pericytes, and Astrocytes
Source: Pharmaceuticals (Basel). 2024 Nov 22;17(12):1567. doi: 10.3390/ph17121567 (PMC11679882; doi:10.3390/ph17121567)

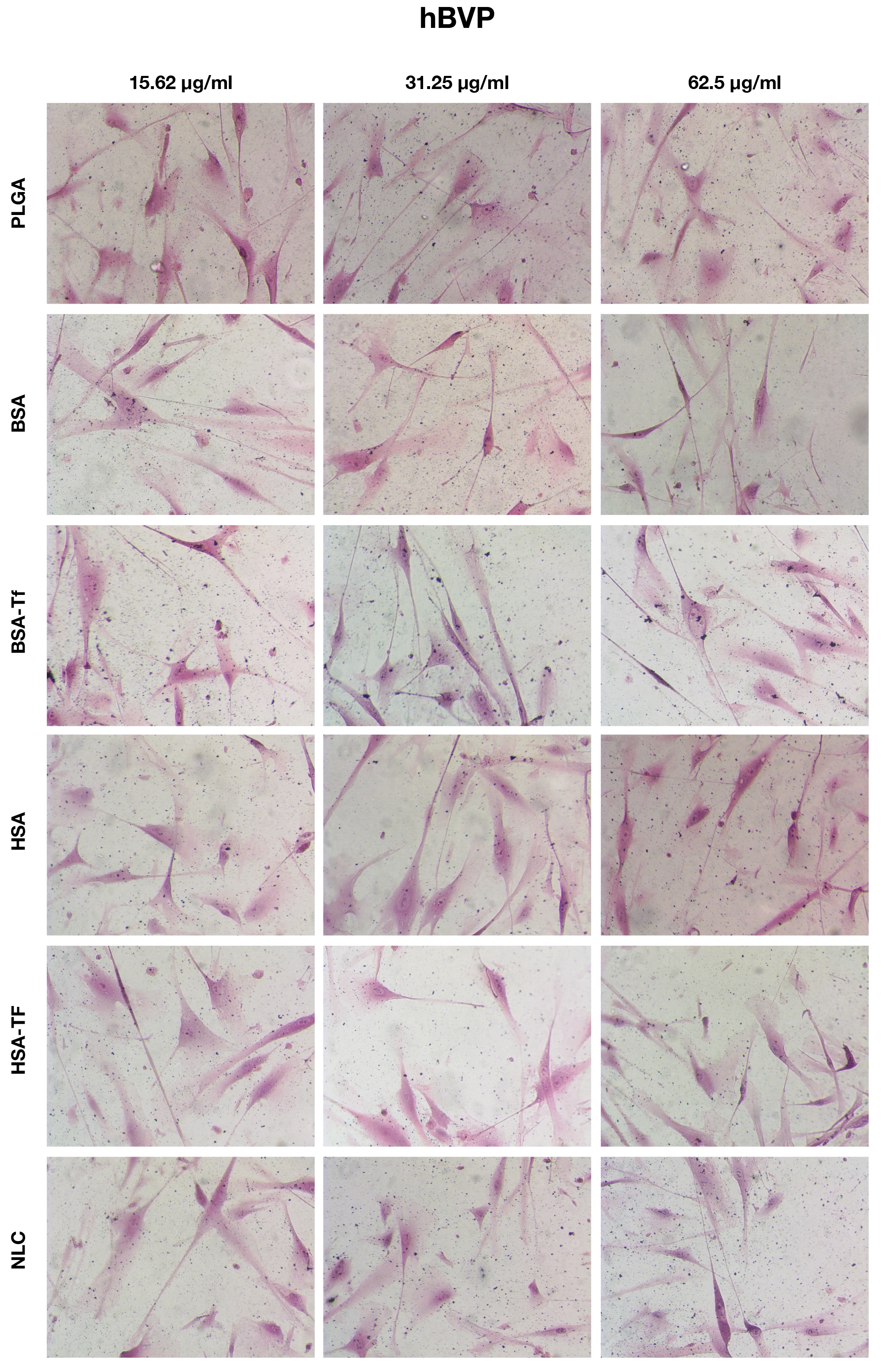

Supplement: Supplementary file 1 [file pharmaceuticals-17-01567-s001.zip › Figure S1.jpeg]

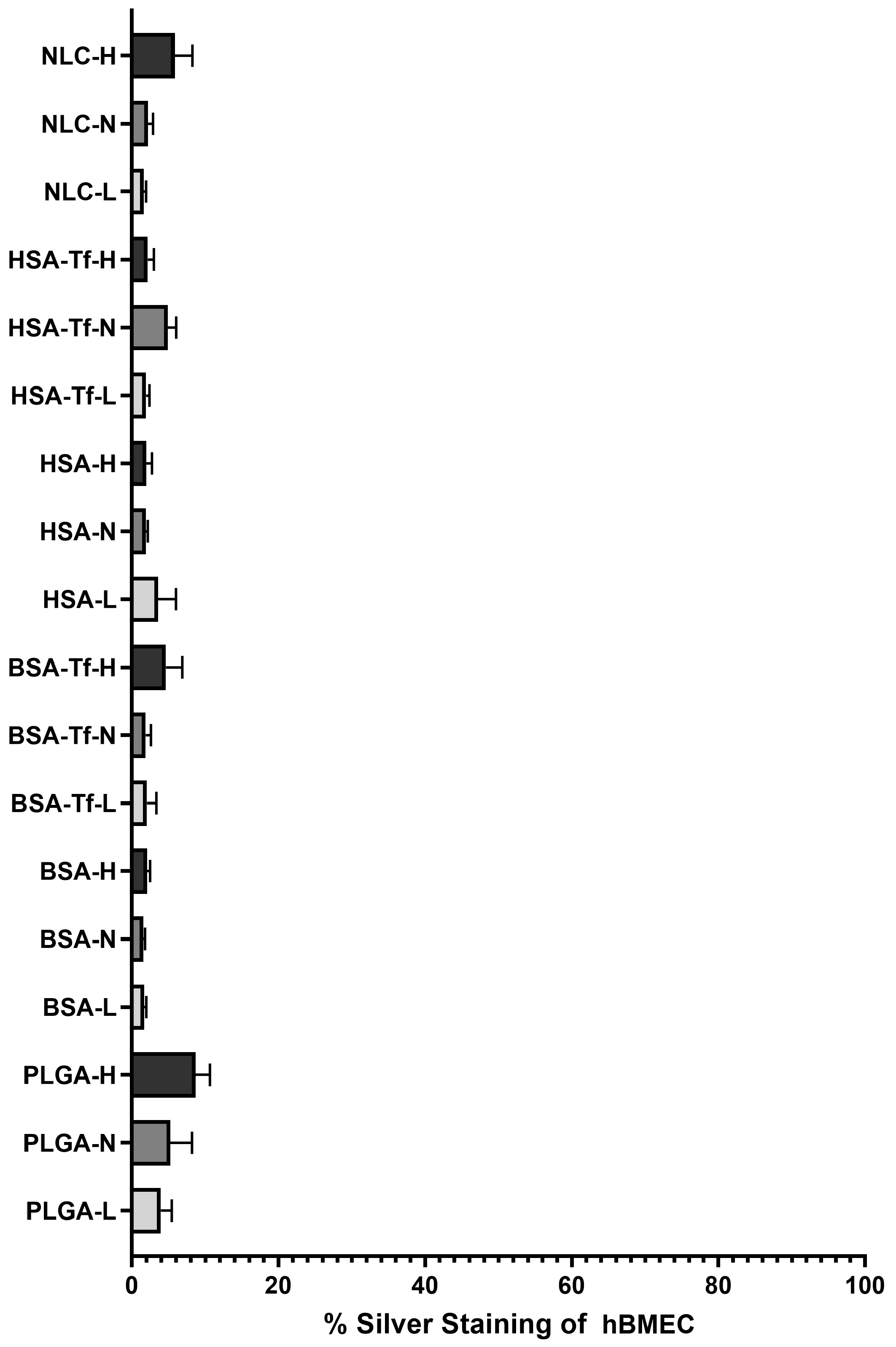

Supplement: Supplementary file 1 [file pharmaceuticals-17-01567-s001.zip › Figure S2.jpeg]

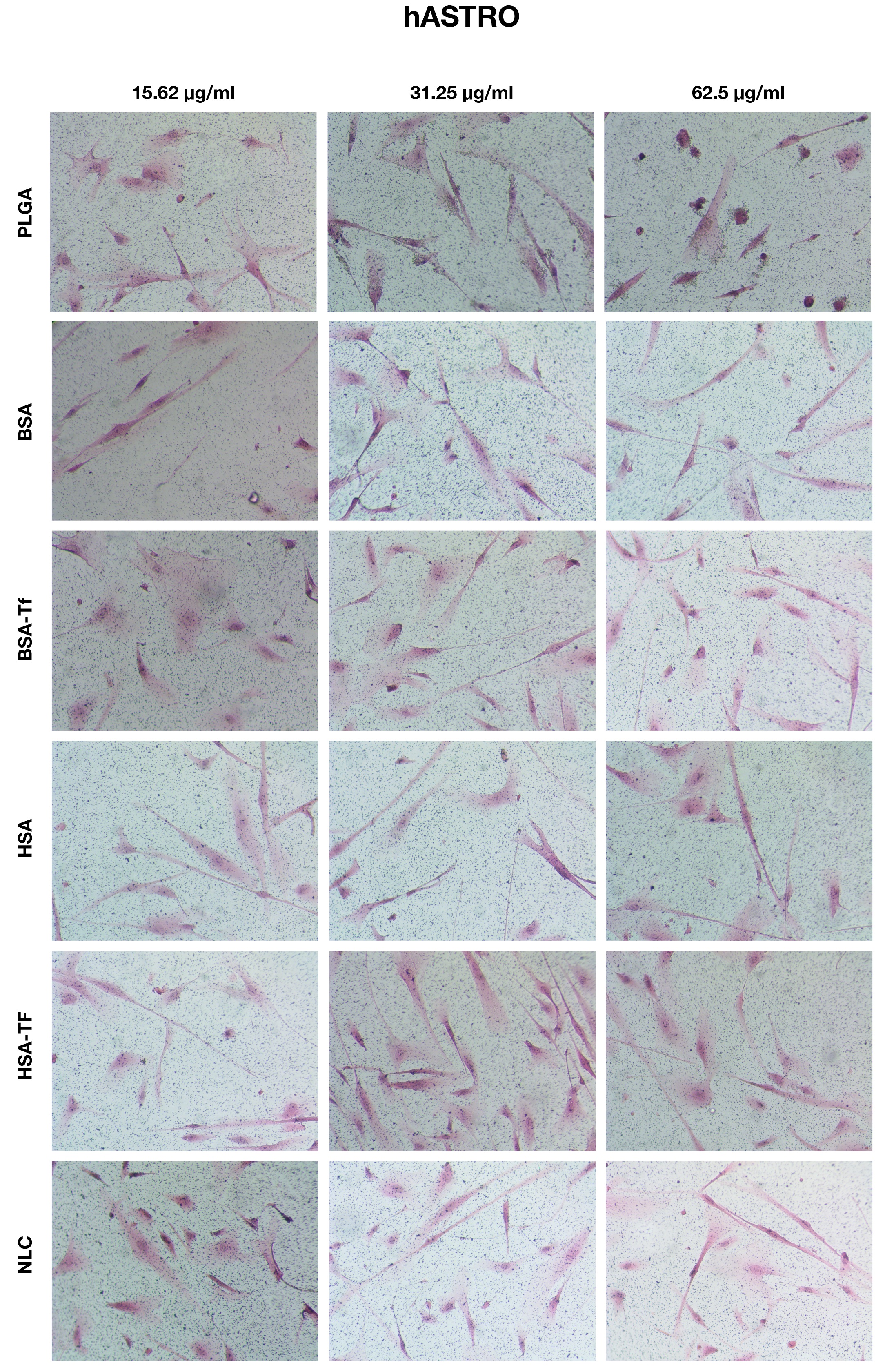

Supplement: Supplementary file 1 [file pharmaceuticals-17-01567-s001.zip › Figure S3.jpeg]

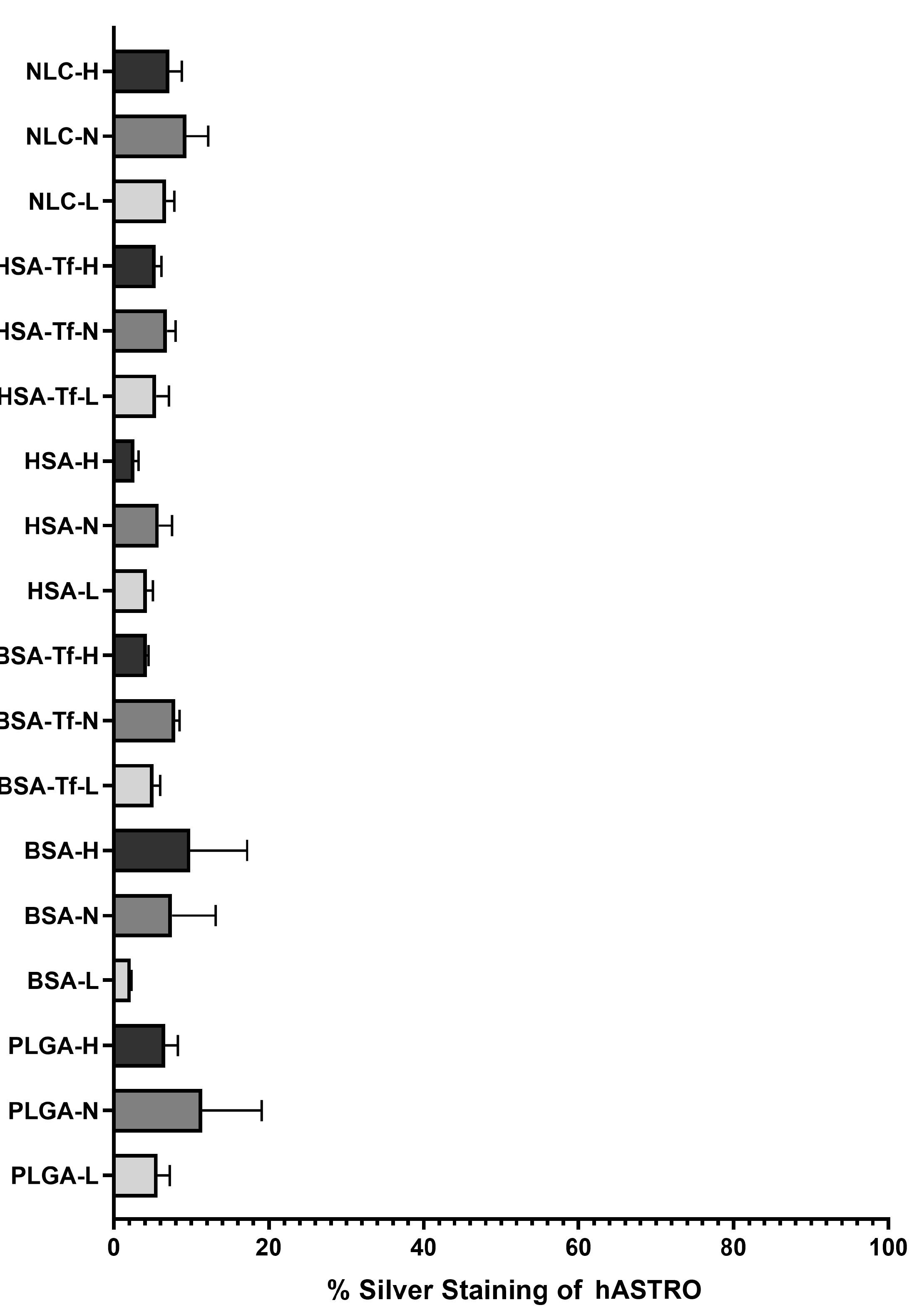

Supplement: Supplementary file 1 [file pharmaceuticals-17-01567-s001.zip › Figure S4.jpeg]
